# Mitochondrial phosphoenolpyruvate carboxykinase promotes tumor growth in estrogen receptor‐positive breast cancer via regulation of the mTOR pathway

**DOI:** 10.1002/cam4.4969

**Published:** 2022-06-27

**Authors:** Hui‐Ping Hsu, Pei‐Yi Chu, Tsung‐Ming Chang, Kuo‐Wei Huang, Wen‐Chun Hung, Shih Sheng Jiang, Hui‐You Lin, Hui‐Jen Tsai

**Affiliations:** ^1^ Department of Surgery National Cheng Kung University Hospital, College of Medicine, National Cheng Kung University Tainan Taiwan; ^2^ Department of Pathology Show Chwan Memorial Hospital Changhua Taiwan; ^3^ National Institute of Cancer Research National Health Research Institutes Tainan Taiwan; ^4^ School of Medicine, College of Medicine Fu Jen Catholic University New Taipei City Taiwan; ^5^ Department of Post‐Baccalaureate Medicine, College of Medicine National Chung Hsing University Taichung Taiwan; ^6^ Department of Medical Laboratory Science College of Medical Science and Technology, I‐Shou University Kaohsiung Taiwan; ^7^ Department of Oncology, National Cheng Kung University Hospital, College of Medicine National Cheng Kung University Tainan Taiwan; ^8^ Department of Internal Medicine, Kaohsiung Medical University Hospital Kaohsiung Medical University Kaohsiung Taiwan

**Keywords:** E2F1, estrogen receptor positive (ER^+^) breast cancer, mitochondrial phosphoenolpyruvate carboxykinase (PEPCK‐M), mTORC1, PCK2

## Abstract

**Background:**

Tumor cells may aberrantly express metabolic enzymes to adapt to their environment for survival and growth. Targeting cancer‐specific metabolic enzymes is a potential therapeutic strategy. Phosphoenolpyruvate carboxykinase (PEPCK) catalyzes the conversion of oxaloacetate to phosphoenolpyruvate and links the tricarboxylic acid cycle and glycolysis/gluconeogenesis. Mitochondrial PEPCK (PEPCK‐M), encoded by *PCK2*, is an isozyme of PEPCK and is distributed in mitochondria. Overexpression of *PCK2* has been identified in many human cancers and demonstrated to be important for the survival program initiated upon metabolic stress in cancer cells. We evaluated the expression status of PEPCK‐M and investigated the function of PEPCK‐M in breast cancer.

**Methods:**

We checked the expression status of PEPCK‐M in breast cancer samples by immunohistochemical staining. We knocked down or overexpressed *PCK2* in breast cancer cell lines to investigate the function of PEPCK‐M in breast cancer.

**Results:**

PEPCK‐M was highly expressed in estrogen receptor‐positive (ER^+^) breast cancers. Decreased cell proliferation and G_0_/G_1_ arrest were induced in ER^+^ breast cancer cell lines by knockdown of *PCK2*. PEPCK‐M promoted the activation of mTORC1 downstream signaling molecules and the E2F1 pathways in ER^+^ breast cancer. In addition, glucose uptake, intracellular glutamine levels, and mTORC1 pathways activation by glucose and glutamine in ER^+^ breast cancer were attenuated by *PCK2* knockdown.

**Conclusion:**

PEPCK‐M promotes proliferation and cell cycle progression in ER^+^ breast cancer via upregulation of the mTORC1 and E2F1 pathways. *PCK2* also regulates nutrient status‐dependent mTORC1 pathway activation in ER^+^ breast cancer. Further studies are warranted to understand whether PEPCK‐M is a potential therapeutic target for ER^+^ breast cancer.

## Graphical abstract

PEPCK‐M was highly expressed in ER^+^ breast cancer patients. PEPCK‐M promotes the proliferation and cell cycle progression of ER^+^ breast cancer via upregulation of the mTORC1 and E2F1 pathways. *PCK2* is also involved in the regulation of mTORC1 pathway activation by nutrition status in ER^+^ breast cancer cells. Further study is warranted to understand whether PEPCK‐M can be a potential target for the treatment of ER^+^ breast cancer.

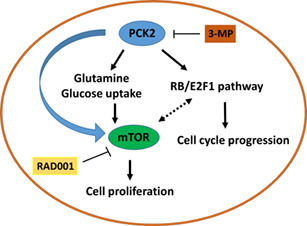

## 1 INTRODUCTION

Breast cancer is the most common cancer in women, with an estimated age‐standardized incidence rate of 46.3 per 100,000 women worldwide in 2018. The mortality rate is high, with an estimated age‐standardized mortality rate of 13 per 100,000 women in 2018.^1^ Furthermore, in Taiwan, the incidence of breast cancer is high, with an age‐standardized incidence rate of 72.99, and the mortality rate was 11.68 per 100,000 women in 2016.^2^ Breast cancer is heterogeneous and usually categorized into different subtypes according to the expression of hormone receptors (the estrogen receptor, ER) and the human epidermal growth factor receptor 2 (HER‐2)/neu receptor. Most early‐stage breast cancers can be cured. However, recurrence or metastasis may develop. The therapeutic goal for metastatic disease is not only to improve survival but also to maintain quality of life.^3^, ^4^ Endocrine therapy, chemotherapy, targeted therapy, and combinations of the above treatments have improved the survival of patients with metastatic breast cancer.^3^, ^4^, ^5^, ^6^, ^7^, ^8^ Other novel agents are also under investigation.

Glycolysis is the predominant mechanism of energy production in most cancer cells, a characteristic termed the Warburg effect.^9^ However, the nutritional conditions in the tumor microenvironment are different from those in normal tissue. In the absence of glucose, tumor cells can utilize other nutrients for cell survival and growth, and the tricarboxylic acid (TCA) cycle may function as the metabolic hub.^10^ Various metabolic enzymes are involved in the survival and progression of cancer cells. Phosphoenolpyruvate carboxykinase (PEPCK), the initial enzyme in gluconeogenesis, catalyzes the conversion of oxaloacetate to phosphoenolpyruvate (PEP) and links the TCA cycle and glycolysis/gluconeogenesis.^10^ There are two PEPCK isozymes. The *PCK1* and *PCK2* genes encode cytosolic PEPCK (PEPCK‐C) and mitochondrial PEPCK (PEPCK‐M), respectively.^11^, ^12^ PEPCK‐C functions primarily in gluconeogenesis.^13^ PEPCK‐M is much less efficient for gluconeogenesis than is PEPCK‐C.^13^ PEPCK‐M has been demonstrated to enhance cell proliferation and respond to stress or nutrient restriction in cancer cells.^14^, ^15^, ^16^, ^17^, ^18^ Overexpression of *PCK2* has been identified in cancer cells in many anatomical sites in humans, including the thyroid, breast, lung, and urinary tract, according to The Cancer Genome Atlas (TCGA) analysis.^16^ Leithner et al. showed that *PCK2* (encoding PEPCK‐M) was overexpressed in lung cancer cells but not in alveolar cells by both mRNA detection and immunohistochemical staining.^17^ The expression of *PCK2* in lung cancer cell lines increases in response to low‐glucose conditions. PEPCK‐M promotes sphere formation and responses to low‐glucose stress in lung cancer cell lines. We previously showed that pancreatic neuroendocrine tumors (pNETs) had differential expression of PEPCK‐M. PEPCK‐M regulates the cell metabolism of pNETs and desensitizes pNETs to mTOR inhibitors.^18^ Mendez‐Lucas et al. showed that nutrient restriction and endoplasmic reticulum stress regulate *PCK2* expression in breast cancer cells. Nutrition restriction also induces apoptosis in breast cancer cells with silencing of *PCK2*.^15^ Breast cancers are heterogeneous, with differentially activated pathways found among different subtypes. Although overexpression of *PCK2* was found in breast cancer, the expression status of PEPCK‐M and the role of *PCK2* in different subtypes of breast cancer are not well understood. Here, we checked the expression status and function of PEPCK‐M in breast cancers to understand whether PEPCK‐M is a potential therapeutic target for breast cancer.

## 2 MATERIALS AND METHODS

### 2.1 Cell lines, plasmids, and reagents

We purchased MCF‐7 and T47D cells from the Bioresource Collection and Research Center (BCRC). The medium and supplements for culture of MCF‐7 cells were Eagle's minimum essential medium (HyClone), 10% fetal bovine serum (FBS), 2 mM L‐glutamine and antibiotics. The medium and supplements for culture of T47D cells were RPMI 1640 medium (HyClone), 10% FBS, 4.5 g/L glucose, 2 mM L‐glutamine, 10 mM HEPES, 1.0 mM sodium pyruvate, and antibiotics. For cell maintenance, the incubator was kept at 37°C with 5% CO_2_. The shRNA sequences targeting *PCK2* and pLKO.1‐null‐T (vector control) were purchased from the National RNAi Core Facility of Academia Sinica. The *PCK2* overexpression plasmid and vector control (EX‐Z2611‐Lv157, pEZ‐Lv157) were obtained from GeneCopoeia. RAD001 was purchased from Selleckchem. 3‐Mercaptopicolinic acid (3‐MP) was purchased from Santa Cruz.

### 2.2 Stable cell clone establishment

Knockdown or overexpression of *PCK2* in cell lines was performed by lentiviral infection. The instructions were provided by the National RNAi Core Facility of Academic Sinica. Western blotting was used to confirm PEPCK‐M expression in the stable cell clones.

### 2.3 Cell proliferation and survival assays

We seeded MCF‐7 cells (2 × 10^4^) or T47D cells (3 × 10^4^) in 24‐well culture plates. The indicated agents were added to the cells for the indicated durations. The proliferation and survival rates of cells under the indicated conditions were determined by a methylene blue colorimetric assay and cell counting. The details of the methylene blue assay, were as described in our previous paper.^18^ Cell counting was performed by trypan blue exclusion technique with a hemocytometer. The proliferation and survival rates under each indicated condition as assessed by the methylene blue method were determined in triplicate, and the experiment was performed twice. Cell counting was performed in quadruplicate.

### 2.4 Western blotting

The details of the western blot analysis were as described in our previous paper.^19^ An antibody against PEPCK‐M (GTX114919) was purchased from GeneTex. Antibodies against E2F1 (sc‐81,257), CDK4 (sc‐260), cyclin D2 (sc‐593) and GAPDH (sc‐32,233) were purchased from Santa Cruz. Antibodies against S6K (#9202), 4EBP‐1 (#9452), phospho‐4EBP‐1^Thr70^ (#9455s), and cyclin D1 (#2926) were purchased from Cell Signaling. An antibody against phospho‐S6K^Thr389^ (MABS82) was purchased from Millipore Group. The quantitative intensity of protein bands was determined with Image J (National Institutes of Health). The expression ratio of the indicated protein to the control protein was defined as the intensity of the indicated protein band divided by the intensity of the control protein band and was further normalized to the intensity of the internal control protein band.

### 2.5 Expression array and gene set enrichment analysis (GSEA)

Gene expression microarray analyses were performed according to a previously described protocol to obtain the differential expression profiles of stable shPCK2‐transfected clones and pLKO.1‐null‐T (vector control)‐transfected clones.^20^ Gene sets or molecular pathways enriched with the differentially expressed genes were analyzed by GSEA using the log2 ratio as the ranking metric, and the ratio was defined as the normalized intensity of a specific gene in shPCK2‐transfected cells divided by that in control cells.^21^

### 2.6 Cell cycle analysis

We cultured cells under the indicated conditions in serum‐free medium for 24 h. The experiments to the compare cell cycle in cells with or without knockdown of *PCK2* or overexpression of *PCK2* were performed with triplicate samples under each indicated condition, and the experiment was performed twice. The experiments to compare cell cycle transitions in cells with or without *PCK2* knockdown or overexpression at different time points were performed in quadruplicate. The experiment to compare cell cycle transitions in MCF‐7 cells treated with different concentrations of 3‐MP at different time points were performed in quadruplicate. The details of cell processing and flow cytometric analysis of the cell cycle were as described in our previous paper,^22^ and the data were analyzed by using ModFit LT 2.0 (Becton Dickinson).

### 2.7 Measurement of glycolysis and mitochondrial oxidative phosphorylation

We seeded MCF‐7 cells (2.0 × 10^4^ cells/well) in XF 24‐well cell culture microplates (Seahorse Bioscience) in 100 μl of growth medium. The procedures and measurement of the extracellular acidification rate (ECAR) and oxygen consumption rate (OCR) of MCF‐7 cells were performed as described in our previous paper.^18^ The ECAR is presented in mpH/min, and the OCR is presented in pmol/min. Each experiment was carried out in duplicate or triplicate.

### 2.8 Measurements of glucose uptake and the intracellular glutamine content

We seeded MCF‐7 cells (1 × 10^5^) infected with vector control or shPCK2 lentivirus in 24‐well plates and then incubated them at 37°C in 5% CO_2_ for 20–24 h. The experiments were performed in triplicate under each condition and each experiment was performed twice. The following day, the medium was removed, and the cells were washed twice with PBS. The cells were cultured in fresh medium at 37°C and 5% CO_2_ for another 24 h, and the intracellular glutamine level and glucose uptake were then measured. For measurement of the intracellular glutamine level, the cells were harvested and the cell lysates were sampled to analyze the intracellular glutamine level by using a glutamine colorimetric assay kit (K556‐100, BioVision, Milpitas, CA) according to the manufacturer's instructions. To measure glucose uptake, we washed the cells with PBS three times and then preincubated them with 100 μl of Krebs–Ringer Phosphate HEPES buffer for 40 min. Then, 10 μl of 10 mM 2‐deoxyglucose (2‐DG) was added to the cells and incubated for 20 min. Then, 180 μl of extraction buffer was added to lyse the cells. The extracted lysates were subjected to freeze‐thaw cycle. The thawed lysates were heated at 85°C for 40 min. Then 20 μl of neutralization buffer was mixed with each cell lysate, and glucose uptake was measured with a glucose uptake colorimetric assay kit (BioVision, Milpitas, CA, K676‐100) in a microplate reader. The total protein concentrations in the cell lysates were determined via the Bradford assay for normalization.

### 2.9 Immunohistochemical (IHC) analysis

A total of 177 patients who were diagnosed with breast cancer and underwent radical resection between January 2007 and December 2012 at National Cheng Kung University Hospital (NCKUH) were enrolled. Patients who received conservative treatment or had other types of breast tumors were excluded. In addition, patients who refused to join the study were excluded. Demographic characteristics, histopathological findings, and clinical outcomes were obtained by conducting a retrospective review of the patient charts. All patients received standard adjuvant therapy according to the guidelines of NCKUH. Formalin‐fixed, paraffin‐embedded sections of breast cancer tissue were obtained from the Human Biobank, Research Center of Clinical Medicine, and the Cancer Data Bank of NCKUH. The study was approved by the Institutional Review Board of NCKUH and the National Health Research Institutes (NHRI) and was performed according to the guidelines and regulations of the NHRI and NCKUH. Formal written informed consent was obtained from each patient.

An anti‐PEPCK‐M antibody (GTX114919) was purchased from GeneTex (Irvine, CA). Four micrometer‐thick paraffin sections were sliced, mounted to slides, and coated with poly‐L‐lysine. Each slide was deparaffinized and rinsed with Tris‐HCl (10 mM, pH 7.4) and sodium chloride (150 mM). Each slide was treated with a solution of methanol and 3% hydrogen peroxide and then placed in citrate buffer (10 mM) in a 100°C heating chamber for 20 min. Then, anti‐PEPCK‐M (1:200) antibody solution was added to each slide for 1 h and incubated at room temperature. After that, the slides were washed with PBS three times. We used an EnVision Detection Systems Peroxidase/DAB, Rabbit/Mouse kit (Dako) to detect bound antibodies and counterstained the slides with hematoxylin. Finally, the slides were evaluated with a microscope (BX50, Olympus). The intensities and area percentages of the signals were evaluated by a board‐certified pathologist. The IHC intensity was scored as follows: “0” for negative staining, “1+” for weak staining, “2+” for moderate staining, and “3+” for strong staining.

### 2.10 Bioinformatics analysis

The cBioPortal database collects multidirectional cancer genomics and proteomics.^23^, ^24^, ^25^ A total of 17 datasets including primary or metastatic breast cancer were selected from cBioPortal. Breast fibroepithelial neoplasms, xenografts of breast cancer, adenoid cystic breast cancer, juvenile papillomatosis, metaplastic breast cancer, and dataset with drug resistance to anti‐HER2 therapy were excluded. The expression level of *PCK2* mRNA was compared with the protein level of ESR1 in reverse‐phase protein arrays (RPPA). The sequencing data of *PCK2* was also recorded, including gain, amplification, shallow deletion, diploid, and genetic variant of uncertain significance (splicing, missense, and truncating).

### 2.11 Statistical analysis

We used SAS statistical software (version 9.4, SAS Institute Inc.) to perform statistical analyses. The distribution of PEPCK‐M in breast cancer tissue was analyzed by the chi‐square test. The OD values measured for cell proliferation or cell survival, the cell counts, the percentages of cells in each cell cycle phase, and the ECAR and OCR values are shown as the means ± standard errors. Cell proliferation or cell cycle alterations between different conditions were compared by the Wilcoxon–rank sum test, with a two‐sided *p* value of less than 0.05 indicating significance. The values of glucose uptake and intracellular glutamine content are shown as the means ± standard errors. Differences in glucose uptake and the intracellular glutamine content between MCF‐7 cells with and without knockdown of *PCK2* were analyzed by the Wilcoxon–rank sum test, with a two‐sided *p* value of less than 0.05 indicating significance. The Bonferroni correction was used to adjust for multiple comparisons.

## 3 RESULTS

### 3.1 PEPCK‐M is differentially expressed in breast tumors

**FIGURE 1:**
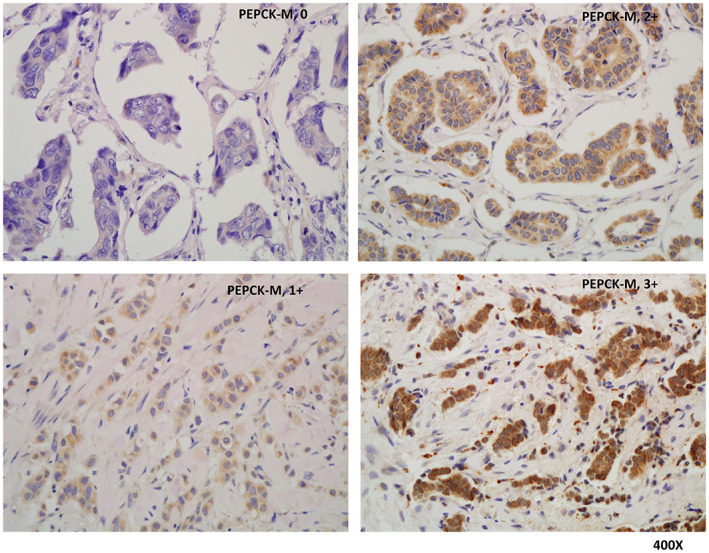
The representative immunohistochemical stainings (0, 1+, 2+, and 3+) of PEPCK‐M in breast cancer patients. 0: left upper; 1+: left lower; 2+: right upper; 3+: right lower.

Because the mRNA expression of *PCK2* was increased in breast cancer according to TCGA analysis, immunohistochemical staining for PEPCK‐M was performed to determine the expression pattern of PEPCK‐M in breast cancer. The expression status of PEPCK‐M in 177 breast cancer patients stratified by HR status, HER‐2/neu expression, and intrinsic subtype is shown in Table 1. The demographic characteristics of these patients are shown in Table S1. Among all patients, a higher percentage (73%) of breast cancer patients exhibited high expression (2+ and 3+) of PEPCK‐M. High PEPCK‐M expression was found in a higher percentage of ER^+^ positive patients (80% with score of 2+ and 3+) than ER^−^ patients (55% with score of 2+ and 3+) (chi‐square test, *p* = 0.004), as shown in Table 1. A lower percentage of patients with the basal‐like intrinsic subtype had high expression of PEPCK‐M (46% with score of 2+ and 3+). Representative results of IHC staining for PEPCK‐M in breast cancer samples are shown in Figure 1.

**TABLE 1:** The expression pattern of PEPCK‐M in breast cancer by ER, PR, HER2/neu status, and intrinsic subtype

	Expression of PEPCK‐M (*N* [%])				*p*‐value^a^
	0	1+	2+	3+	
Patient number (%)	4 (2%)	45 (25%)	93 (53%)	35 (20%)	
Estrogen receptor					
Negative	1 (2%)	23 (44%)	19 (36%)	10 (19%)	0.004
Positive	3 (2%)	22 (18%)	74 (60%)	25 (20%)	
Progesterone receptor					
Negative	3 (3%)	31 (30%)	50 (49%)	19 (18%)	0.303
Positive	1 (1%)	14 (19%)	43 (58%)	16 (22%)	
HER2/neu					
Negative	3 (3%)	34 (28%)	60 (49%)	24 (20%)	0.647
Positive	1 (2%)	11 (20%)	32 (59%)	10 (19%)	
Intrinsic subtype					
Luminal A	1 (2%)	10 (23%)	21 (49%)	11 (26%)	0.049
Luminal B1	2 (4%)	10 (19%)	32 (59%)	10 (19%)	
Luminal B2	0	3 (10%)	21 (70%)	6 (20%)	
HER2/neu‐enriched	1 (4%)	8 (33%)	11 (46%)	4 (17%)	
Basal‐like	0	14 (54%)	8 (31%)	4 (15%)	

^a^ Chi‐square test.

### 3.2 *PCK2* promotes the proliferation and metabolism of ER ^+^ breast cancer cells

**FIGURE 2:**
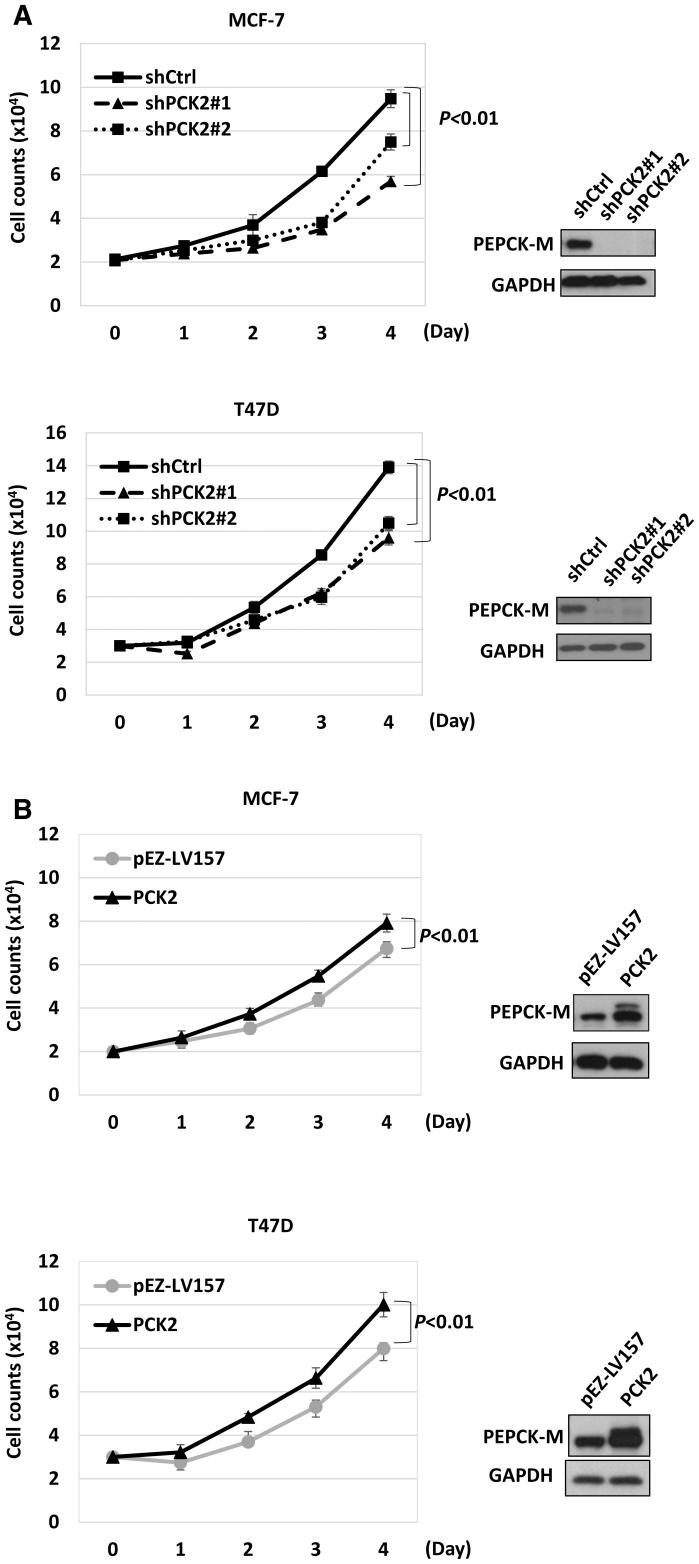
The promoting effect of *PCK2* on cell proliferation in ER^+^ breast cancer. (A) The daily cell count of MCF‐7 (upper) and T47D (lower) with and without *PCK2* knockdown. (B) The daily cell count of MCF‐7 (upper) and T47D (lower) with and without *PCK2* overexpression. pEZ‐LV157 is the vector control.

Because higher expression of PEPCK‐M was found in ER^+^ breast cancer samples than in ER^−^ breast cancer samples and ER^+^ breast cancer is the most common subtype,^3^ we evaluated the function of *PCK2* in ER^+^ breast cancer. We knocked down *PCK2* by using a lentiviral approach in the ER^+^ breast cancer cell lines MCF‐7 and T47D and established stable clones of these cell lines with and without knockdown of *PCK2*. Cell proliferation in both cell lines was significantly attenuated in cells in which *PCK2* was knocked down using two different *PCK2‐*shRNAs (shPCK2#1 and shPCK2#2) compared with the two cell lines transduced with pLKO.1‐null‐T (shCtrl), as shown by cell counting (Figure 2A) and the methylene blue assay (Figure S1A). Furthermore, we overexpressed *PCK2* in these two cell lines. Overexpression of *PCK2* promoted proliferation in both cell lines (Figure 2B and Figure S1B). Because *PCK2* is associated with the metabolism in pNETs according to our previous study,^18^ we measured the ECAR and OCR, which are indicators of glycolysis and mitochondrial function, respectively, in MCF‐7 cells. The ECAR and OCR of MCF‐7 cells were reduced by the knockdown of *PCK2* (Figure S2). These results confirm that *PCK2* promotes the proliferation of ER^+^ breast cancer cells and suggest that *PCK2* promotes glycolysis and mitochondrial oxidative phosphorylation in ER^+^ breast cancer cells.

### 3.3 *PCK2* promotes cell cycle progression in ER ^+^ breast cancer cells via the regulation of cell cycle molecules

**FIGURE 3:**
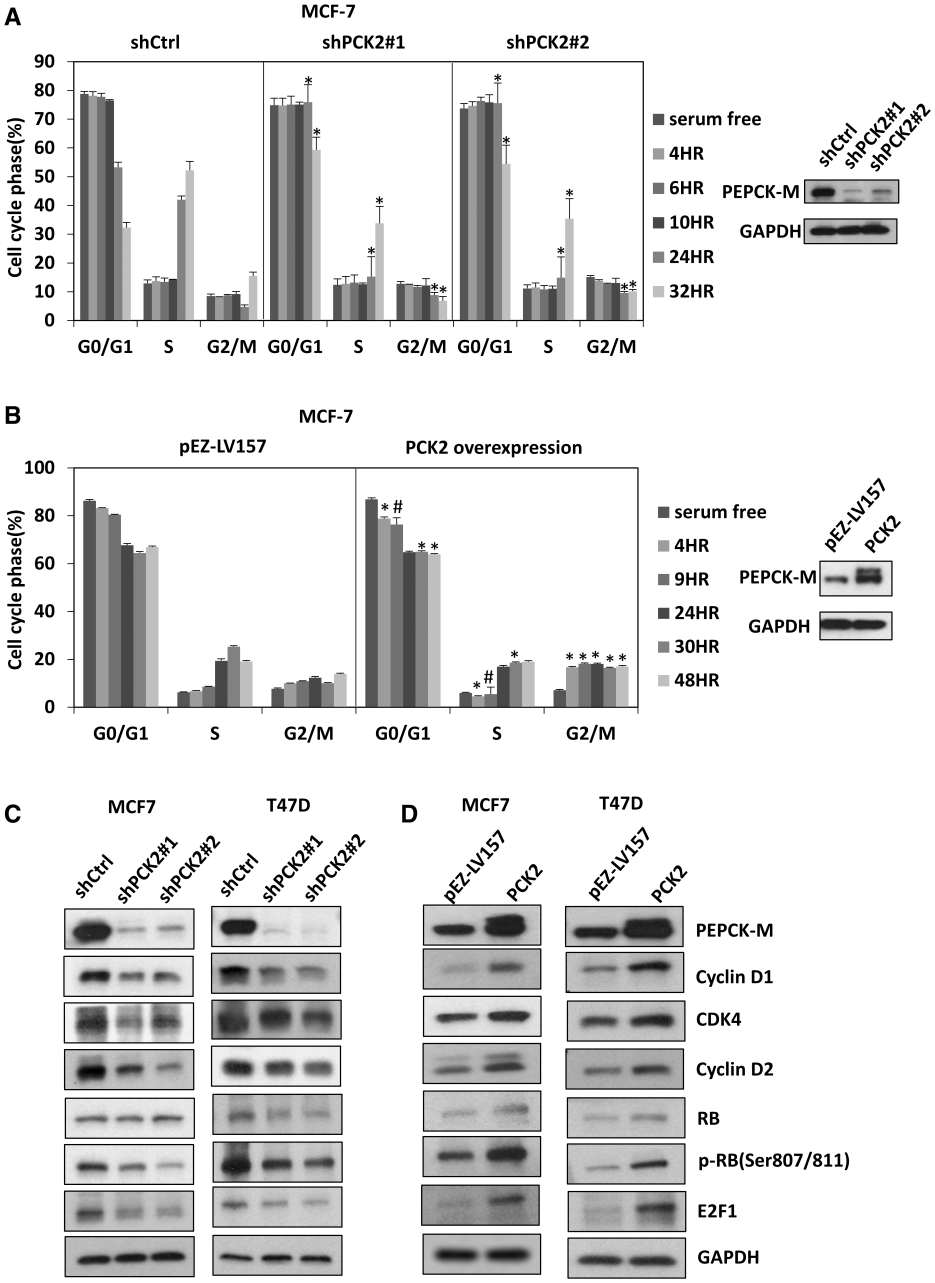
*PCK2* promotes cell cycle progression in ER^+^ breast cancer via regulation of cell cycle molecules. (A) The cell cycle analysis of MCF‐7 with and without *PCK2* knockdown in a time course. *p = 0.03. (B) The cell cycle analysis of MCF‐7 with and without *PCK2* overexpression in a time course. *p = 0.03; #*p* = 0.014. (C) The cell cycle‐regulating molecules in MCF‐7 (left) and T47D (right) with and without *PCK2* knockdown after 48 h incubation. (D) The cell cycle‐regulating molecules in MCF‐7 (left) and T47D (right) with and without *PCK2* overexpression after 48 h incubation. pEZ‐LV157 is the vector control.

To explore the underlying roles of *PCK2* in breast cancer cell proliferation, we assessed the effect of *PCK2* on cell cycle progression using flow cytometric analysis. Knockdown of *PCK2* resulted in accumulation of ER^+^ MCF‐7 and T47D cells in G_0_/G_1_ phase, accompanied by a reduction in S‐phase cells, as shown in Figure S3A,B. In contrast, overexpression of *PCK2* in ER^+^ MCF‐7 cells induced accumulation of cells in S phase, accompanied by a reduction in the proportion of G_2_/M‐phase cells (Figure S3A). In addition, we synchronized MCF‐7 cells to G_0_/G_1_ phase by serum starvation to evaluate the effect of *PCK2* on cell cycle progression. The G_1_/S and S/G_2_/M transitions tended to be delayed in MCF‐7 cells with *PCK2* knockdown compared with cells without *PCK2* knockdown (shCtrl), as shown in Figure 3A (*p* = 0.03). In contrast, *PCK2* overexpression in MCF‐7 cells promoted cell cycle progression from G_1_ to G_2_/M phase compared with that in cells without *PCK2* overexpression (vector control), as shown in Figure 3B. A similar pattern of cell cycle progression was observed in T47D cells with *PCK2* knockdown (Figure S4A) or *PCK2* overexpression compared with control cells (Figure S4B). The G_0_/G_1_/S transition tended to be delayed by knockdown of *PCK2* in T47D cells compared with control cells (*p* = 0.03). The G_0_/G_1_/S and S/G_2_/M transitions were faster in T47D cells overexpressing *PCK2* than in vector control cells. We checked a panel of cell cycle‐regulating proteins associated with the G_0_/G_1_/S transition. The protein levels of cyclin D1, cyclin D2, CDK4, RB, phosphorylated RB, and E2F1 were decreased in both MCF‐7 and T47D cells with *PCK2* knockdown, as shown in Figure 3C. In contrast, the levels of these molecules were increased in MCF‐7 and T47D cells overexpressing *PCK2*, as shown in Figure 3D. These results indicate that *PCK2* promotes the G_1_/S transition via the regulation of cell cycle molecules.

### 3.4 *PCK2* promotes cell cycle progression by regulating the mTOR pathway

**FIGURE 4:**
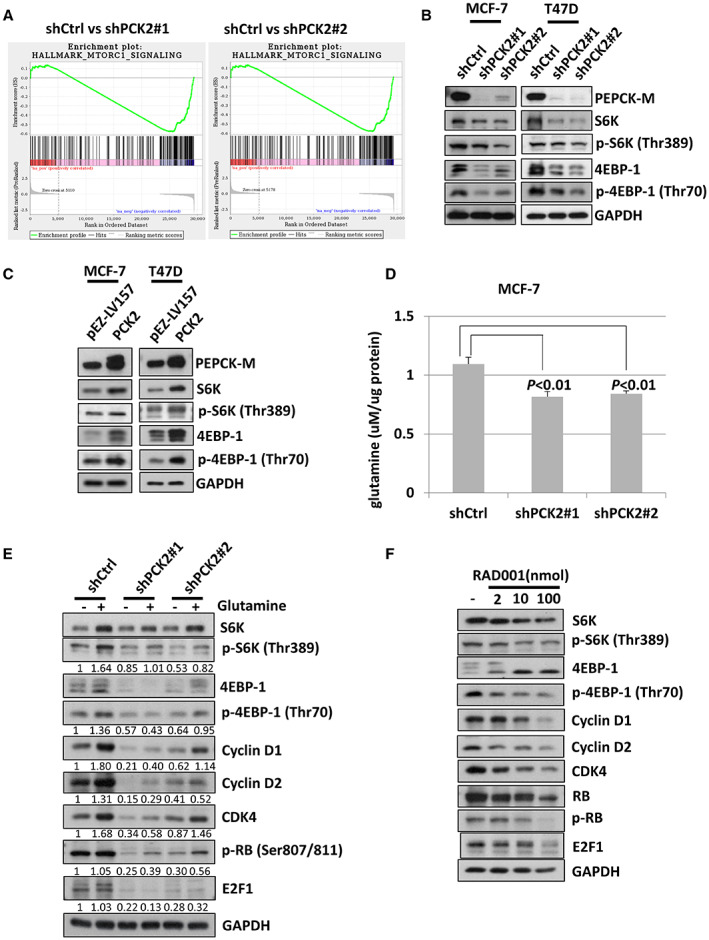
*PCK2* promotes cell cycle progression in ER^+^ breast cancer cells by regulating mTOR pathway. (A) The gene sets (mTORC1) was negatively enriched in MCF‐7 cells with *PCK2* knockdown. (B) The expression status of downstream signals of mTORC1, S6K, and 4EBP‐1 in MCF‐7 (left) and T47D (right) cells with and without *PCK2* knockdown after 48 h incubation. (C) The expression status of downstream signals of mTORC1, S6K, and 4EBP‐1 in MCF‐7 (left) and T47D (right) cells with and without *PCK2* overexpression after 48 h incubation. pEZ‐LV157 is the vector control. (D) The intracellular glutamine level in MCF‐7 cells with and without *PCK2* knockdown checked after 24 h incubation. (E) The expression of downstream signals of mTORC1 and cell cycle‐regulating molecules in MCF‐7 cells with and without *PCK2* knockdown and with or without supplement of glutamine for 24 h. (F) The expression of downstream signals of mTORC1 and cell cycle associated molecules in MCF‐7 cells treated with indicated doses of RAD001 for 24 h.

To evaluate the potential molecular pathways regulated by *PCK2*, we performed microarray analyses of gene expression in MCF‐7 cells with and without *PCK2* knockdown. GSEA found that mTORC1‐related genes were less enriched in MCF‐7 cells with *PCK2* knockdown than in control cells (Figure 4A). Figure 4B shows that the expression of 4EBP‐1 and S6K, downstream targets of mTORC1, was decreased in MCF‐7 cells with *PCK2* knockdown. Similar results were observed in T47D cells (Figure 4B). In contrast, the expression of 4EBP‐1 and S6K was increased in MCF‐7 and T47D cells overexpressing *PCK2*, as shown in Figure 4C.

Because mTORC1 is an important regulator of protein synthesis, cell growth and metabolism in response to amino acids and growth factors,^26^, ^27^ we evaluated whether *PCK2* affects the metabolism of ER^+^ breast cancer cells and investigated its association with mTORC1 signaling. We measured the intracellular glutamine level in MCF‐7 cells with and without *PCK2* knockdown, as shown in Figure 4D. The intracellular glutamine level was reduced in MCF‐7 cells with *PCK2* knockdown. We also evaluated the signaling molecules downstream of mTORC1 in MCF‐7 cells with and without glutamine supplementation. The protein levels of S6K, phosphorylated S6K, 4EBP‐1, and phosphorylated 4EBP‐1 in MCF‐7 cells with or without *PCK2* knockdown were lower in the absence of glutamine (Figure 4E). Under glutamine supplementation, the levels of these proteins were increased. However, the increases in these proteins were only partially rescued in MCF‐7 cells with *PCK2* knockdown compared with cells without *PCK2* knockdown (shCtrl). In the absence of glutamine, the levels of cyclin D1, cyclin D2, and CDK4, which are cell cycle regulators required for the G_1_/S transition, were decreased in MCF‐7 cells with or without *PCK2* knockdown. The increases in these molecules were also impaired in MCF‐7 cells with *PCK2* knockdown and supplemented with glutamine. In addition, we evaluated the levels of phosphorylated RB and E2F1, which are important factors for cell cycle progression, in MCF‐7 cells. The protein levels of E2F1 and phosphorylated RB were decreased by knockdown of *PCK2* and were lower in the absence of glutamine supplementation. Similarly, the increases in E2F1 and phosphorylated RB induced by glutamine supplementation were only partially rescued in MCF‐7 cells with *PCK2* knockdown compared with cells without *PCK2* knockdown (shCtrl). The response of *PCK2* to glucose deprivation in MCF‐7 cells was similar to that under glutamine deprivation. Glucose uptake was reduced in MCF‐7 cells with *PCK2* knockdown (Figure S5A). In the absence of glucose, the expression levels of downstream molecules of mTORC1, that is, S6K, phosphorylated S6K, 4EBP‐1, and phosphorylated 4EBP‐1, as well as those of cell cycle regulators were low in MCF‐7 cells with or without *PCK2* knockdown (Figure S5B). These levels were also only partially rescued by supplementation of glucose in MCF‐7 cells with *PCK2* knockdown compared with cells without *PCK2* knockdown (shCtrl) (Figure S5B).

To delineate the correlations of cell cycle regulators with mTORC1 activation, we treated MCF‐7 cells with different concentrations of the mTOR inhibitor RAD001. The effects on the signaling molecules downstream of mTORC1 and the cell cycle regulators cyclin D1, cyclin D2, CDK4, RB, phosphorylated RB, and E2F1 are shown in Figure 4F. The protein levels of all cell cycle regulators were decreased by the addition of RAD001 in a concentration‐dependent manner. The proliferation of MCF‐7 cells over a 4‐day period was reduced by the addition of RAD001 (Figure S6). These results demonstrate that *PCK2* regulates cell cycle regulators through a mTORC1‐dependent pathway. Taken together, the results clarify that *PCK2* promotes cell cycle progression by regulating mTORC1 activation and the responses of ER^+^ breast cancer cells to glutamine and glucose supplementation.

### 3.5 Treatment with the PEPCK inhibitor 3‐MP reduces mTORC1 activity and induces G_0_ /G_1_ ‐phase arrest

**FIGURE 5:**
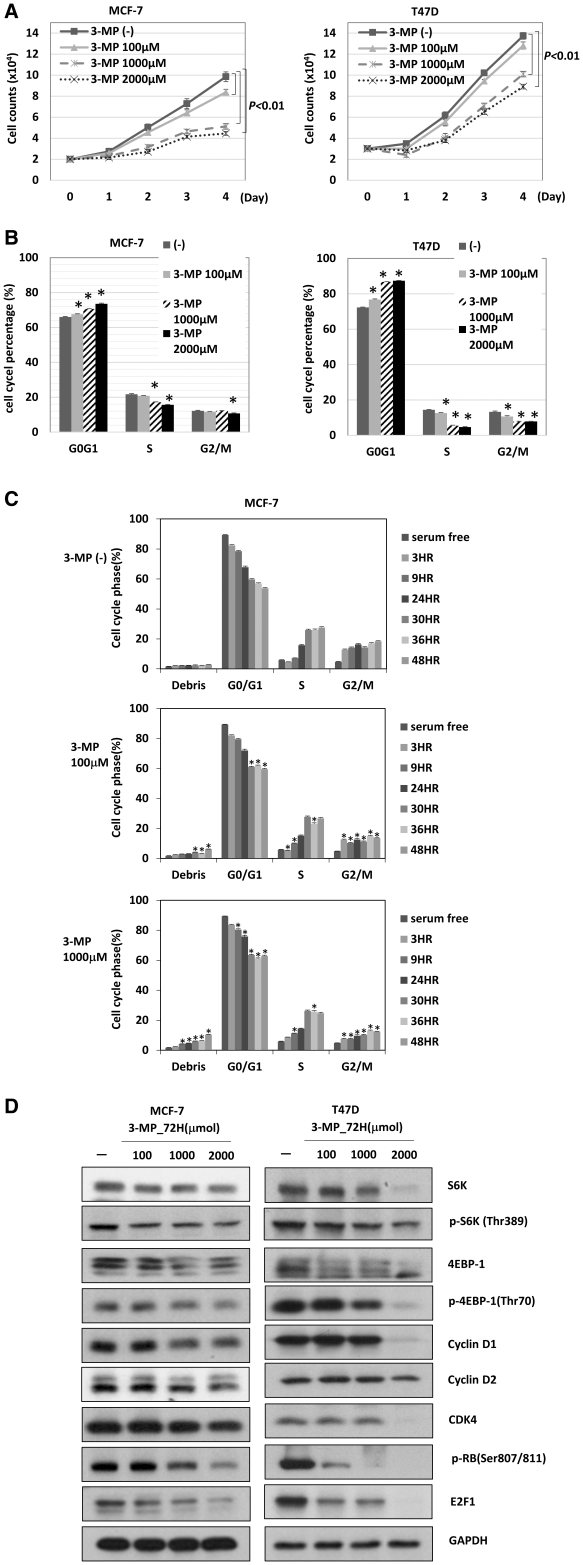
The effect of PEPCK inhibitor, 3‐MP, in ER^+^ breast cancer. (A) The cell count of MCF‐7 (left) and T47D (right) cells treated with various dose of 3‐MP for 4 days. (B) The cell cycle analysis of MCF‐7 (left) and T47D (right) treated with 3‐MP for 72 h. *p = 0.03. (C) The transition of cell cycle of MCF‐7 cells treated with 3‐MP for indicated dose and duration. *p = 0.03. (D) The expression of downstream signals of mTORC1 and cell cycle associated molecules in MCF‐7 (left) and T47D (right) cells treated with different doses of 3‐MP for 72 h.

To validate the effect of *PCK2* on cell proliferation and the cell cycle, we treated MCF‐7 and T47D cells with the PEPCK inhibitor 3‐MP and evaluated its effect on cell proliferation and the cell cycle. The proliferation rates in both cell lines were significantly reduced by 3‐MP in a concentration‐dependent manner, as shown in Figure 5A and Figure S7. Cell cycle analysis showed that 3‐MP tended to induce a concentration‐dependent increase in the proportion of G_0_/G_1_‐phase cells and decreases in S‐ and G_2_/M‐phase cells in both MCF‐7 and T47D cells, particularly in T47D cells (Figure 5B). The transitions from G_0_/G_1_ to G_2_/M phase in MCF‐7 cells and the transition from G_0_/G_1_ phase to S phase in T47D cells also tended to be delayed by 3‐MP (Figure 5C and Figure S8). We checked the expression status of mTORC1 downstream targets and cell cycle regulators in MCF‐7 and T47D cells treated with 3‐MP. Concentration‐dependent decreases in the protein levels of S6K, phosphorylated S6K, 4EBP‐1, phosphorylated 4EBP‐1, E2F1, phosphorylated RB, CDK4, cyclin D1, and cyclin D2 were found in cells treated with 3‐MP, as shown in Figure 5D. These results show that PEPCK‐M is potentially a therapeutic target for ER^+^ breast cancer.

## 4 DISCUSSION

We showed that PEPCK‐M was differentially expressed in different subtypes of breast cancer, with high expression in ER^+^ breast cancer. ER^+^ breast cancer is the most common subtype of breast cancer, accounting for 60%–80% of breast cancers.^3^ Approximately 30% of these patients develop recurrence with metastatic disease and approximately 5%–10% are diagnosed with Stage IV disease.^3^ It is mandatory to develop treatment for prolonging the survival and maintaining the quality of life of patients with advanced ER^+^ breast cancer. In this study, we showed that *PCK2* promotes the proliferation of ER^+^ breast cancer cells via upregulation of the mTORC1 and RB/E2F1 axes, which affects cell cycle progression. ER may activate ER signaling via genomic or nongenomic regulation of gene expression through estrogen binding or various intracellular signaling events.^28^, ^29^ In addition, we observed an association of high PEPCK‐M expression with ER^+^ breast cancer. However, it is unknown whether ER can regulate *PCK2*. The mRNA level of *PCK2* was positively correlated with the protein expression level of ESR1 according to the public database cBioPortal, which contains 10,811 samples from 10,211 patients in 17 studies, as shown in Figure S9.^23^, ^24^, ^25^ The mechanistic implication of the correlation between PEPCK‐M and ER expression needs further investigation. In addition, high expression of PEPCK‐M was found in ER^−^, HER2/neu^+^, HER2/neu^−^, and basal‐like breast cancers, although in different percentages of patients. The results demonstrate the heterogeneity of breast cancer even within the same subtype and suggest diverse mechanisms of regulation of PEPCK‐M expression in breast cancers. Furthermore, study is needed to understand the mechanism of the differential expression of PEPCK‐M in different subtypes of breast cancers.

mTOR is a serine/threonine kinase with an important role in integrating intracellular and extracellular growth signals, the regulation of cellular metabolism, protein synthesis, cell growth, homeostasis, survival, and autophagy.^26^, ^27^, ^30^ Activation of the mTORC1 pathway is commonly observed in ER^+^ breast cancer due to genetic (mutation of genes encoding receptor tyrosine kinases or downstream oncogenes, loss‐of‐function of tumor suppressor genes) or nongenetic factors (increased levels of extracellular growth factors, long‐term estrogen deprivation).^31^, ^32^ PEPCK was demonstrated to promote the activation of mTORC1 in the colon adenocarcinoma cancer cell line Colon205. Knockdown of PEPCK suppressed the proliferation of Colon205 cells.^14^ Previously, we showed the decreased proliferation and downregulation of mTORC1‐related gene sets (*BCAT1*, *FAM129A*, *WARS*, and *IDH1*) in pNET cells with *PCK2* knockdown.^18^ In our current study, we also showed downregulation of mTORC1‐related gene sets in ER^+^ MCF‐7 cells with *PCK2* knockdown. In addition, the levels of downstream signaling molecules of mTORC1, that is, 4EBP‐1 and S6K, were significantly reduced by knockdown of *PCK2* in ER^+^ MCF‐7 and T47D cells. The results suggest that *PCK2* promotes the proliferation of ER^+^ breast cancer cells via activation of the mTORC1 pathway, as it does in other cancer types.

**FIGURE 6:**
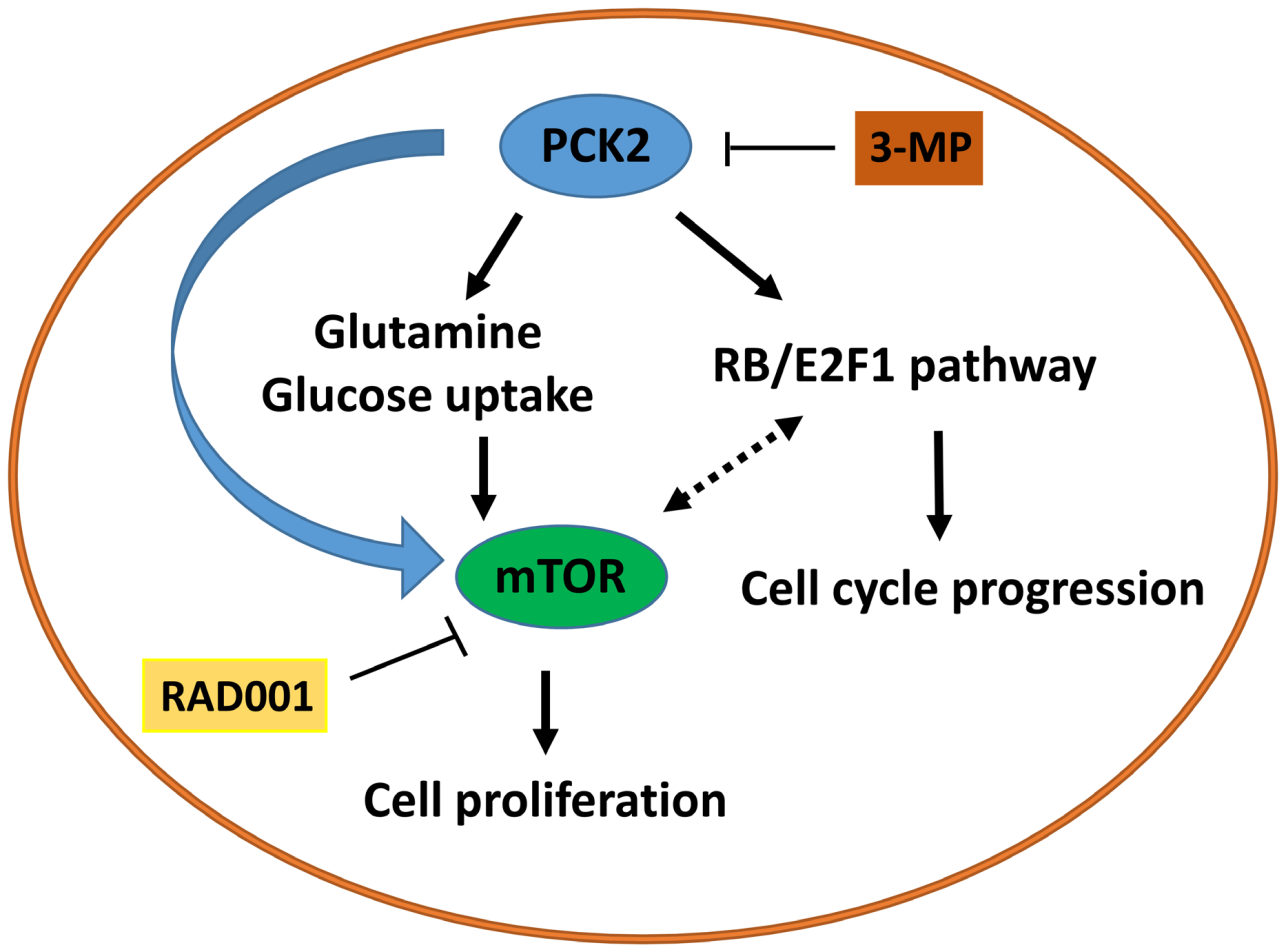
The putative model of regulation of intracellular glutamine and glucose uptake, mTORC1 and RB/E2F1 axes in ER^+^ breast cancer cells by *PCK2*. *PCK2* may reduce intracellular glutamine level and glucose uptake of ER^+^ breast cancer cells. *PCK2* may activate mTORC1 and RB/E2F1 axes to promote cell proliferation and progression of cell cycle. There is association between mTORC1 and RB/E2F1 axes. The mTORC1 activation by glutamine or glucose supplementation may be impaired by suppression of *PCK2*.

We also found that *PCK2* promoted cell cycle progression, particularly the G_1_/S transition, and induced upregulation of E2F1, cyclin D1, cyclin D2, and CDK4 in ER^+^ breast cancer cells. E2F1 is an important transcription factor that regulates the progression of the cell cycle into S phase.^33^ In addition, E2F1 has been shown to regulate cell growth by activating mTORC1 signaling by enhancing v‐ATPase activity and promoting the translocation of mTORC1 into lysosomes.^34^, ^35^ Ladu et al. also showed that transgenic mice overexpressing E2F1 exhibit high mTORC1 activity.^36^ Almacellas et al. showed that E2F1 induces aerobic glycolysis by enhancing PFKFB3 expression, leading to the activation of mTORC1.^37^ These studies demonstrate that E2F1 participates in mTORC1 activation. Furthermore, Michaloglou et al. showed that the mTORC1/2 inhibitor AZD2014 inhibits the phosphorylation of RB and cyclin D1, which is consistent with the reduced phosphorylation of S6K and 4EBP‐1 observed in ER^+^ MCF‐7 and HCC‐1428 cells.^38^ The expression of a panel of E2F‐dependent gene transcription factors, including E2F1, was reduced by AZD2014.^38^ Their results suggest that the CDK‐RB‐E2F pathway can be modulated by mTORC1/2. In the current study, our results support the association of E2F1 and mTORC1 in cell growth and cell cycle progression. In addition, the reductions in the expression of RB, E2F1 and cell cycle‐associated molecules in MCF‐7 cells by mTOR inhibitor treatment further demonstrates the association of mTORC1 with the RB/E2F1 axes in our study. Therefore, *PCK2* promotes cell proliferation and cell cycle progression by regulating mTORC1 and E2F1 in ER^+^ breast cancer. However, the causal effects of E2F1 and mTORC1 mediated by *PCK2* need further investigation. The putative model of *PCK2*‐mediated regulation of the mTORC1 and RB/E2F1 axes is shown in Figure 6.

*PCK2* has been shown to be involved in the regulation of metabolism and drug resistance. PEPCK was shown to promote glycolysis in Colon205 cells and attenuate the sensitivity of Colon205 cells to rapamycin.^14^ In our previous study, we have found that *PCK2* attenuates the sensitivity of pNET cells to mTOR inhibitors. In addition, *PCK2* suppresses glycolysis but promotes mitochondrial oxidative phosphorylation in pNET cells.^18^ In our current study, *PCK2* promoted mTORC1 pathway activation but did not affect the sensitivity of ER^+^ breast cancer cells to an mTOR inhibitor (data not shown). *PCK2* increased the ECAR and OCR in ER^+^ breast cancer cells, suggesting that *PCK2* regulates glycolysis and mitochondrial oxidative phosphorylation in ER^+^ breast cancer cells. *PCK2* regulates drug resistance and the metabolism in pNET cells was not the same as that in ER^+^ breast cancer cells, suggesting a differential function of *PCK2* by cancer type.

PEPCK‐M has been demonstrated to participate in the supportive adaptation of cancer cells under stress.^15^, ^17^ Leithner et al. showed a decrease in PEPCK activity in lung cancer cells with *PCK2* knockdown. Compared with parental cells, lung cancer cells with *PCK2* knockdown cultured under low‐glucose conditions exhibited increased apoptosis. However, the increased apoptosis in lung cancer cells with *PCK2* knockdown was not observed when the cells were cultured under high‐glucose conditions.^17^ Mendez‐Lucas et al. showed that restriction of nutrients (amino acids, including glutamine, arginine, lysine, methionine, and cysteine) or treatment with inducers of endoplasmic reticulum stress (thapsigargin and tunicamycin) upregulated the mRNA expression of *PCK2* in various cancer cell lines—MCF‐7, HeLa, HCT116, and NIH‐3T3Kras. MCF‐7 cells with *PCK2* knockdown developed an increase in apoptosis under glutamine deprivation compared with that in cells without *PCK2* knockdown.^15^ Cancer cells can grow under low‐glucose conditions. Glucose deprivation stimulates reprogramming of the TCA cycle to promote cancer cell proliferation in a glucose‐independent manner. PEPCK may play a cataplerotic role to convert oxaloacetate to PEP and then to pyruvate for use in the TCA cycle.^39^ Vincent et al. showed that glutamine maintains TCA cycle metabolism and the level of PEP, a glycolytic intermediate, in A549 cells under glucose deprivation. PEPCK‐M is required for this metabolic change under glucose deprivation. The production of PEP from glutamine was increased in A549 and H1299 cells in response to glucose deprivation. However, the increased conversion of PEP from glutamine was reduced when *PCK2* was knocked down in these two cell lines.^16^ Glutamine‐derived PEP under low‐glucose conditions can be a source for the biosynthesis of serine and glycine. The biosynthesis of serine and glycine derived from glutamine under glucose depletion was reduced after knockdown of *PCK2*. These results demonstrate that PEPCK‐M can regulate metabolic adaptation and enable glucose‐independent proliferation of cancer cells in a low‐glucose environment via glutamine metabolism.^16^ Glutamine, the most abundant amino acid in the blood, is required for mTORC1 activation and is the rate‐limiting substrate in mTORC1 activation.^40^, ^41^ Long‐term complete deprivation of intracellular glutamine was shown to inhibit mTORC1 (as determined by S6K phosphorylation) in HeLa, U2OS, and HEK293A cells and in TSC2^−/−^ MEFs.^40^ The addition of glutamine to the cells upon glutamine withdrawal activated mTORC1 (increased level of phosphorylated S6K).^40^, ^41^ In our current study, we cannot demonstrate how PEPCK‐M regulates the metabolism of glucose and glutamine. However, we showed that glucose uptake and the intracellular glutamine level were reduced in MCF‐7 cells with *PCK2* knockdown. In addition, the activation of mTORC1 signaling and the cell cycle regulators E2F1/RB, cyclin D1, cyclin D2, and CDK4 by glucose and glutamine were impaired in MCF‐7 cells with *PCK2* knockdown compared with those without *PCK2* knockdown. These results suggest that *PCK2* not only reduces glucose uptake and the glutamine level but also desensitizes cells to the effect of glucose and glutamine on mTORC1 pathway activation.

This study has several strengths. First, we showed the differential expression of PEPCK‐M in different subtypes of breast cancer. Second, we demonstrated that *PCK2* regulates the mTORC1 and RB/E2F1 axes and promotes cell proliferation and cell cycle progression in ER^+^ breast cancer. Third, we demonstrated that *PCK2* affects mTORC1 activation upon nutritional stress in ER^+^ breast cancer. Fourth, this study suggests that PEPCK‐M is a potential therapeutic target for ER^+^ breast cancer treatment. There are several limitations of this study. First, the mechanism of high expression of PEPCK‐M in ER^+^ breast cancer was not delineated. Second, how PEPCK‐M regulates glutamine and glucose metabolism in ER^+^ breast cancer was not investigated. Third, an animal study was not conducted because we failed to engraft ER^+^ breast cancer cells in a xenograft mouse model.

In conclusion, this study found that PEPCK‐M is highly expressed in ER^+^ breast cancer patients, which is a novel finding. PEPCK‐M promotes proliferation and cell cycle progression in ER^+^ breast cancer cells via upregulation of the mTORC1 and RB/E2F1 axes. *PCK2* is also involved in the regulation of mTORC1 pathway activation by nutrient status in ER^+^ breast cancer cells. The high expression of PEPCK‐M and its effect on ER^+^ breast cancer imply the role of PEPCK‐M in cell growth and adaptation to nutrient restriction. Furthermore, studies are warranted to understand whether PEPCK‐M can be a potential therapeutic target for ER^+^ breast cancer.

## AUTHORS' CONTRIBUTIONS

Hui‐Ping Hsu collected the samples, analyzed and interpreted the data; Pei‐Yi Chu did a pathology check and analyzed the data; Kuo‐Wei Huang and Tsung‐Ming Chang designed and performed the experiments; Hui‐You Lin performed the experiments; Wen‐Chun Hung and Shih Sheng Jiang analyzed and interpreted the data; Hui‐Jen Tsai proposed and supervised the study, analyzed and interpreted the data, and wrote the manuscript.

## FUNDING INFORMATION

This study was supported by Ministry of Science and Technology, 108‐2314‐B‐400‐024‐ and 109‐2314‐B‐400‐032‐MY2 and National Health Research Institutes, CA‐108‐PP‐18.

## CONFLICT OF INTEREST

All authors declared no financial conflicts of interests.

## ETHICS APPROVAL

The study was approved by the Institutional Review Board of National Health Research Institutes (NHRI) and National Cheng Kung University Hospital (NCKUH) and was performed according to their guidelines and regulations of NHRI and NCKUH.

## Supporting information


Figure S1

Figure S2

Figure S3

Figure S4

Figure S5

Figure S6

Figure S7

Figure S8

Figure S9

Table S1

## Data Availability

The data that support the findings of this study are available on request from the corresponding author.
