# Supplementary figures and images for: Mitochondrial phosphoenolpyruvate carboxykinase promotes tumor growth in estrogen receptor‐positive breast cancer via regulation of the mTOR pathway
Source: Cancer Med. 2022 Jun 27;12(2):1588–601. doi: 10.1002/cam4.4969 (PMC9883444; doi:10.1002/cam4.4969)

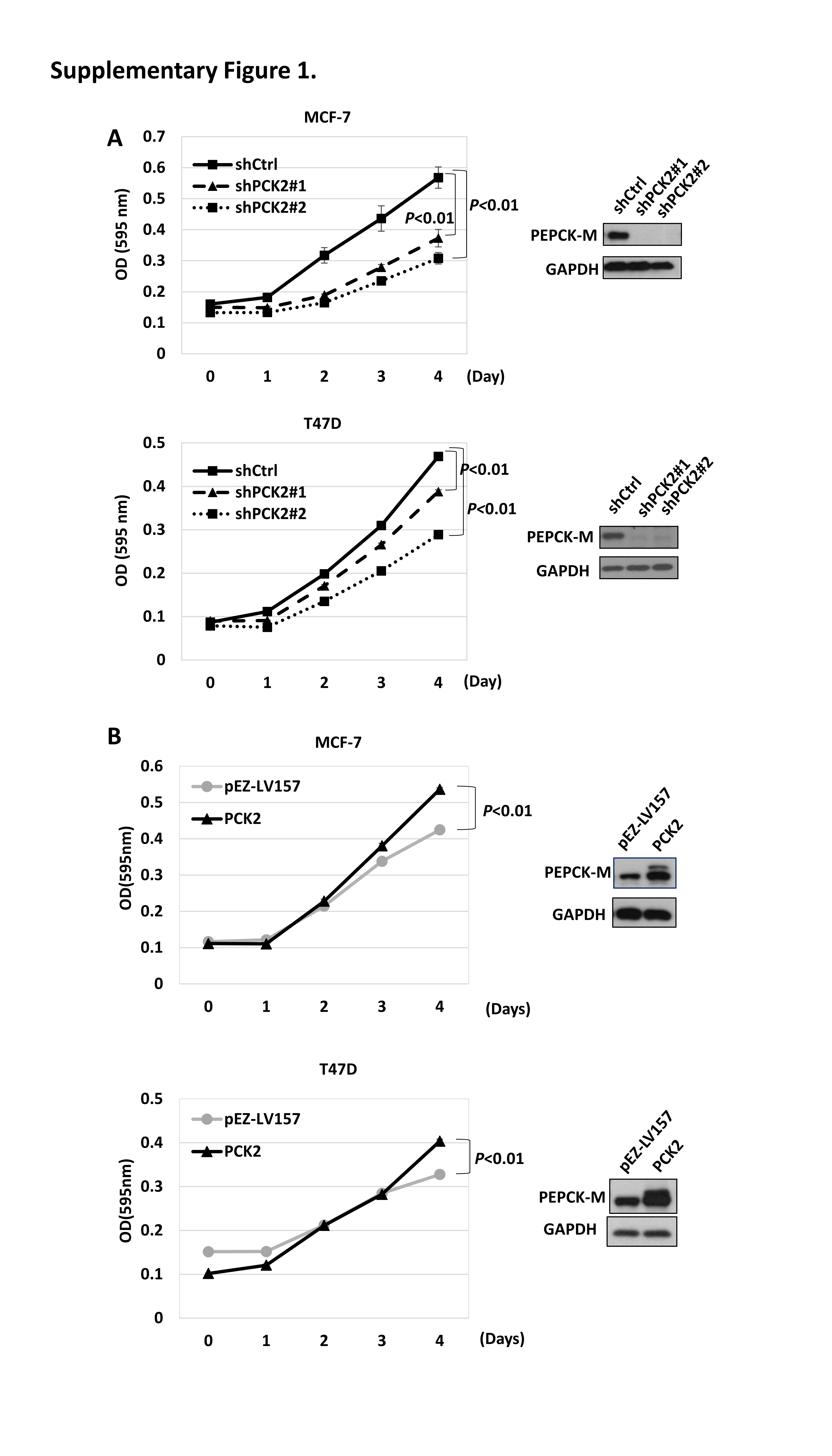

Supplement: Supplementary file 1 — Figure S1 [file CAM4-12-1588-s010.tif]

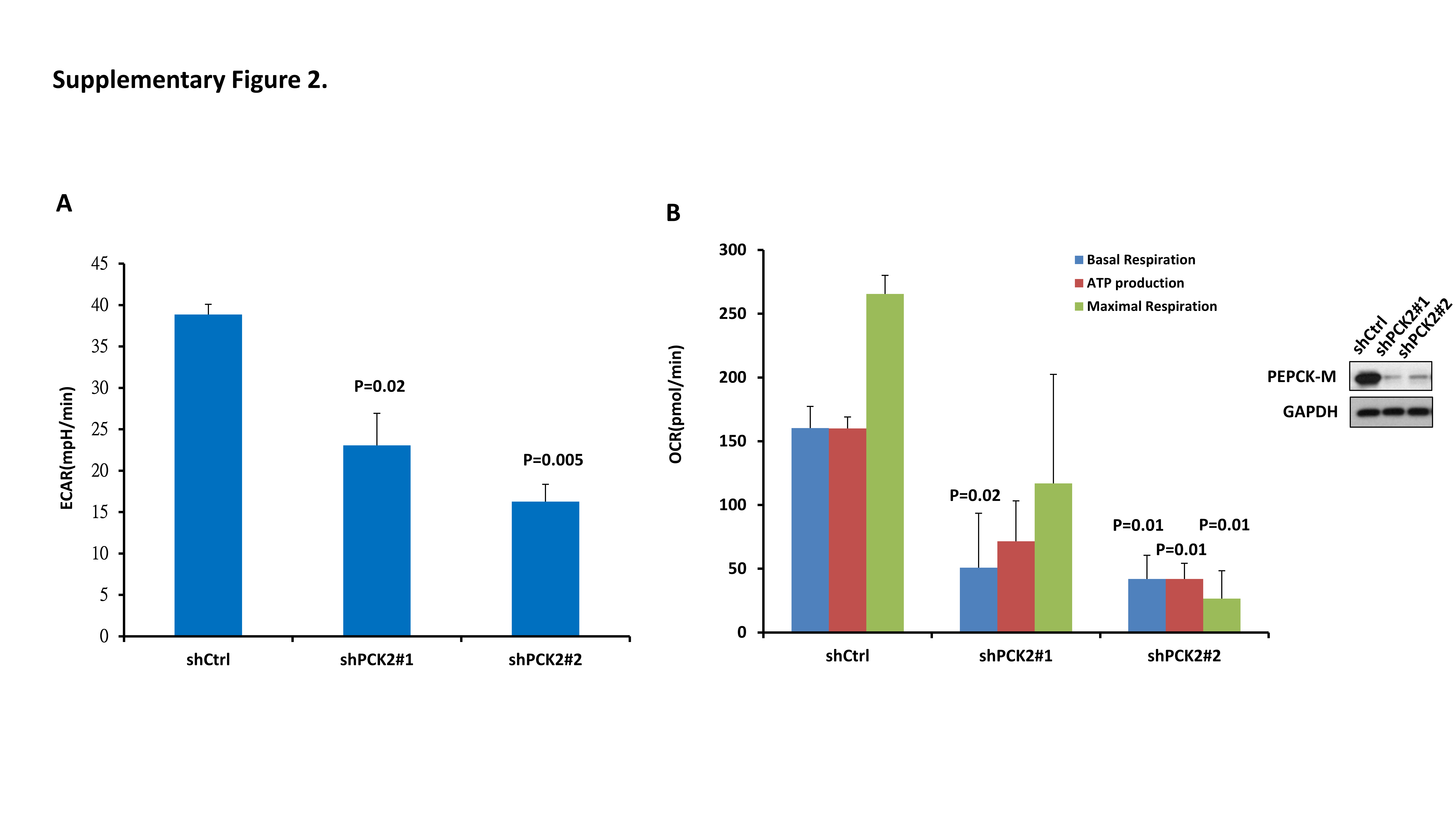

Supplement: Supplementary file 2 — Figure S2 [file CAM4-12-1588-s007.tif]

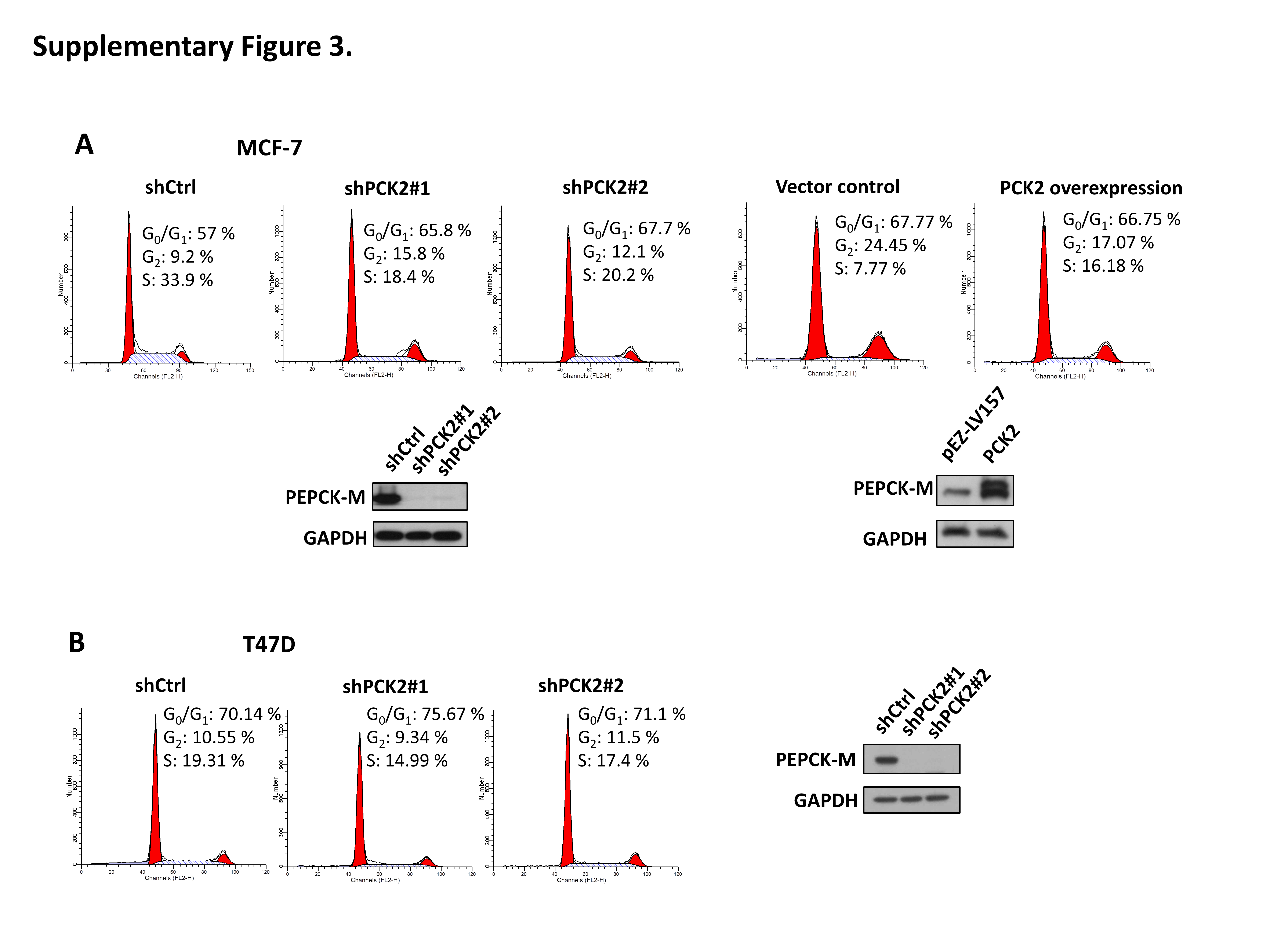

Supplement: Supplementary file 3 — Figure S3 [file CAM4-12-1588-s006.tif]

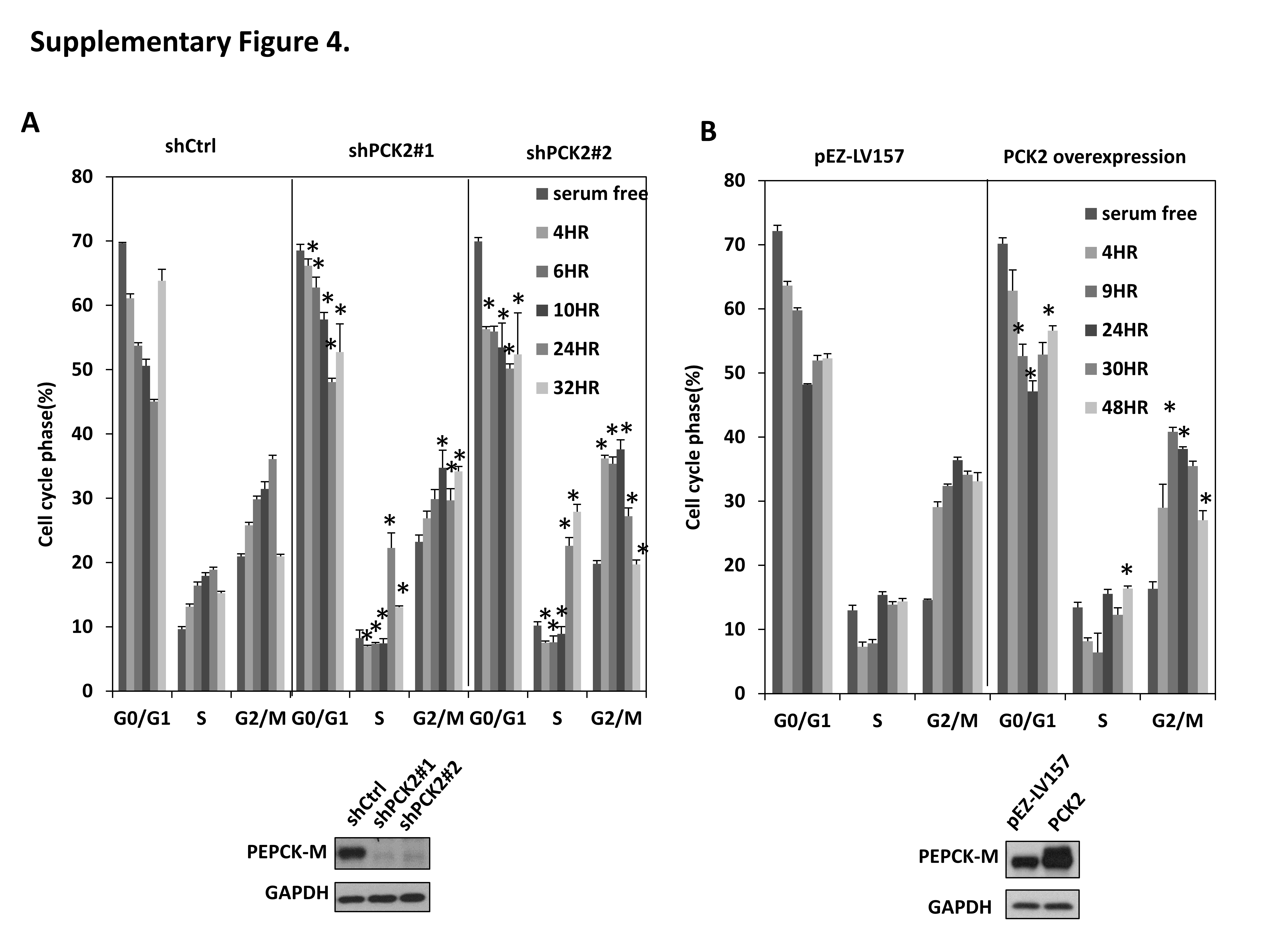

Supplement: Supplementary file 4 — Figure S4 [file CAM4-12-1588-s003.tif]

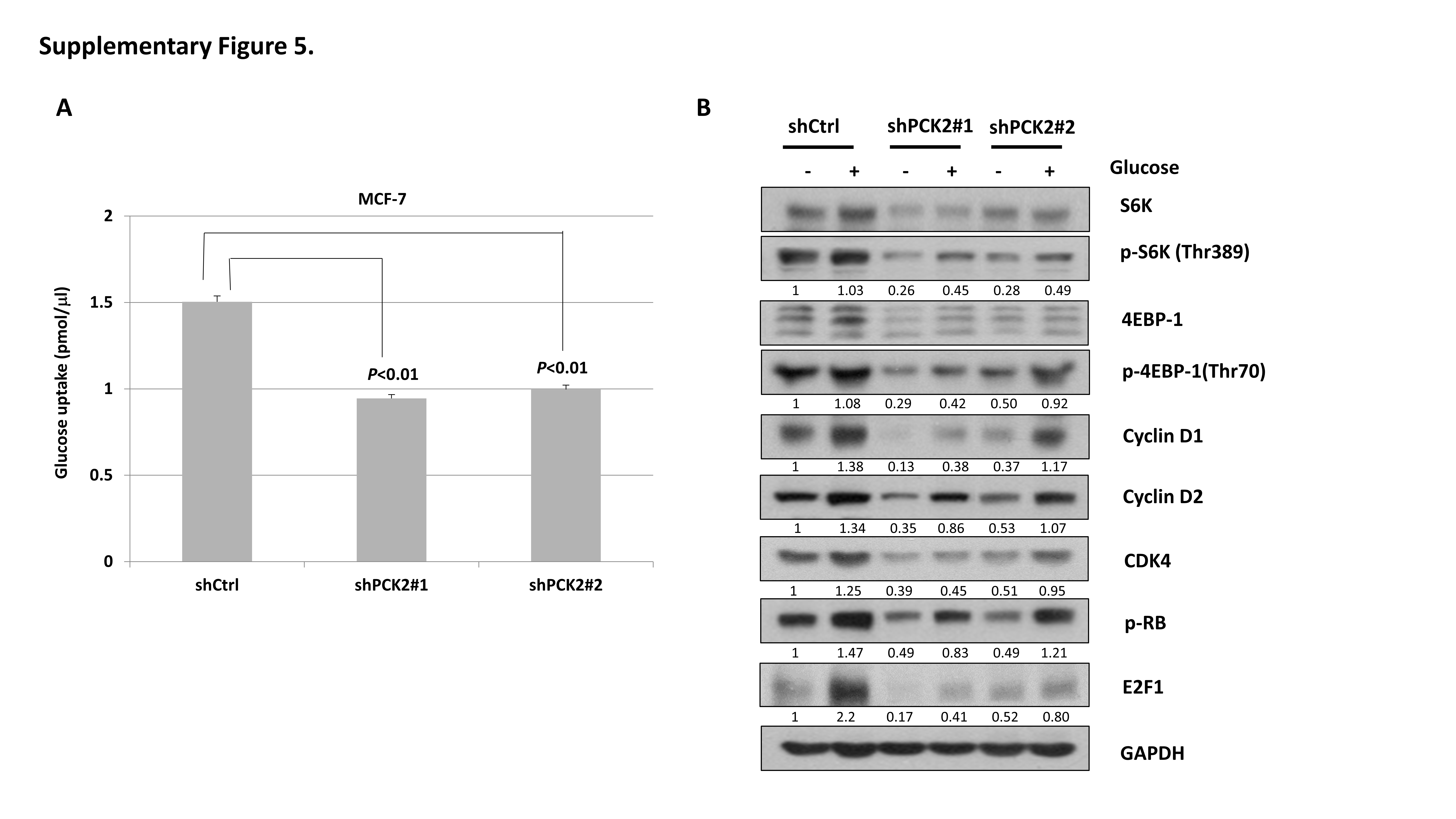

Supplement: Supplementary file 5 — Figure S5 [file CAM4-12-1588-s001.tif]

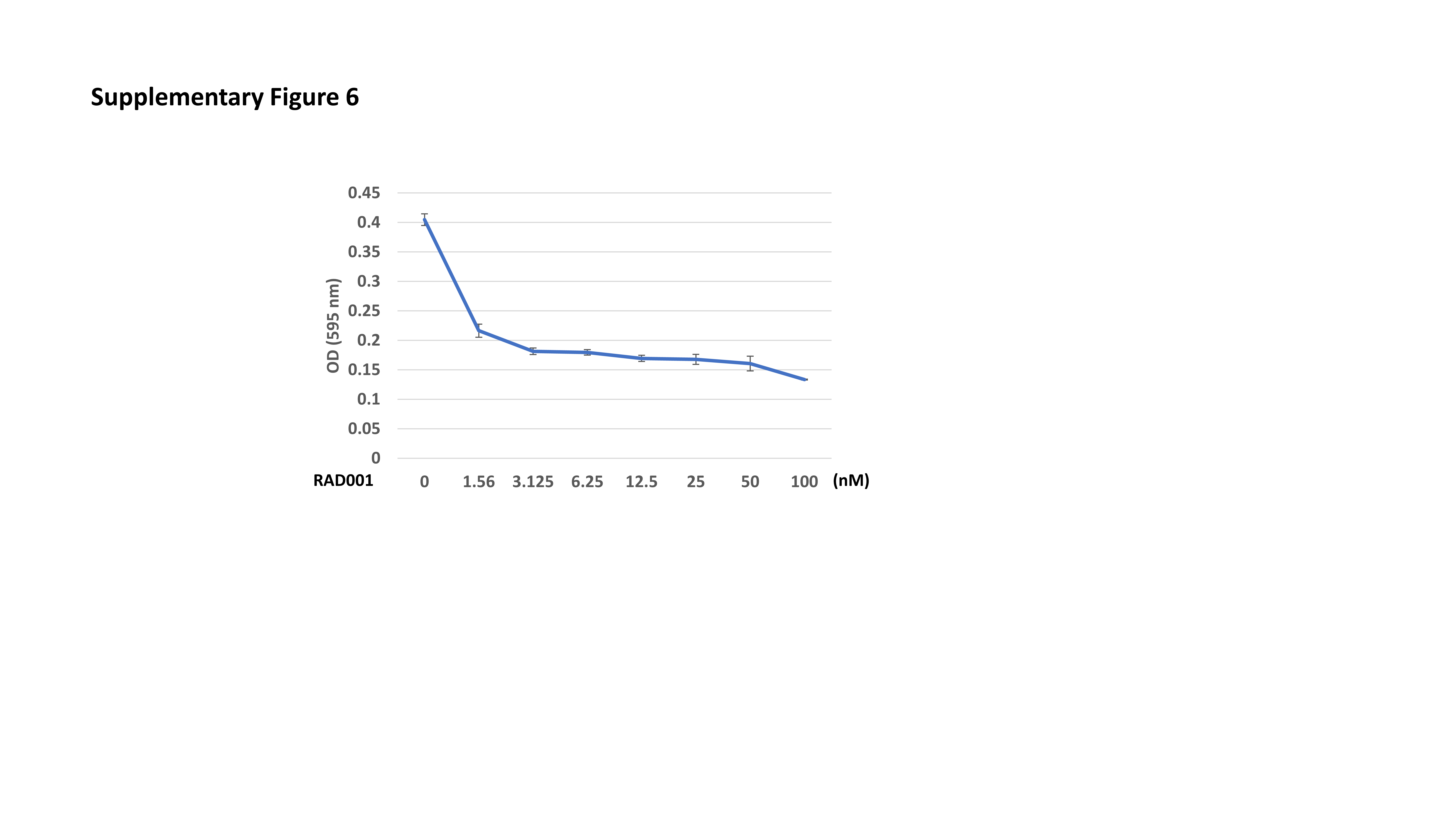

Supplement: Supplementary file 6 — Figure S6 [file CAM4-12-1588-s005.tif]

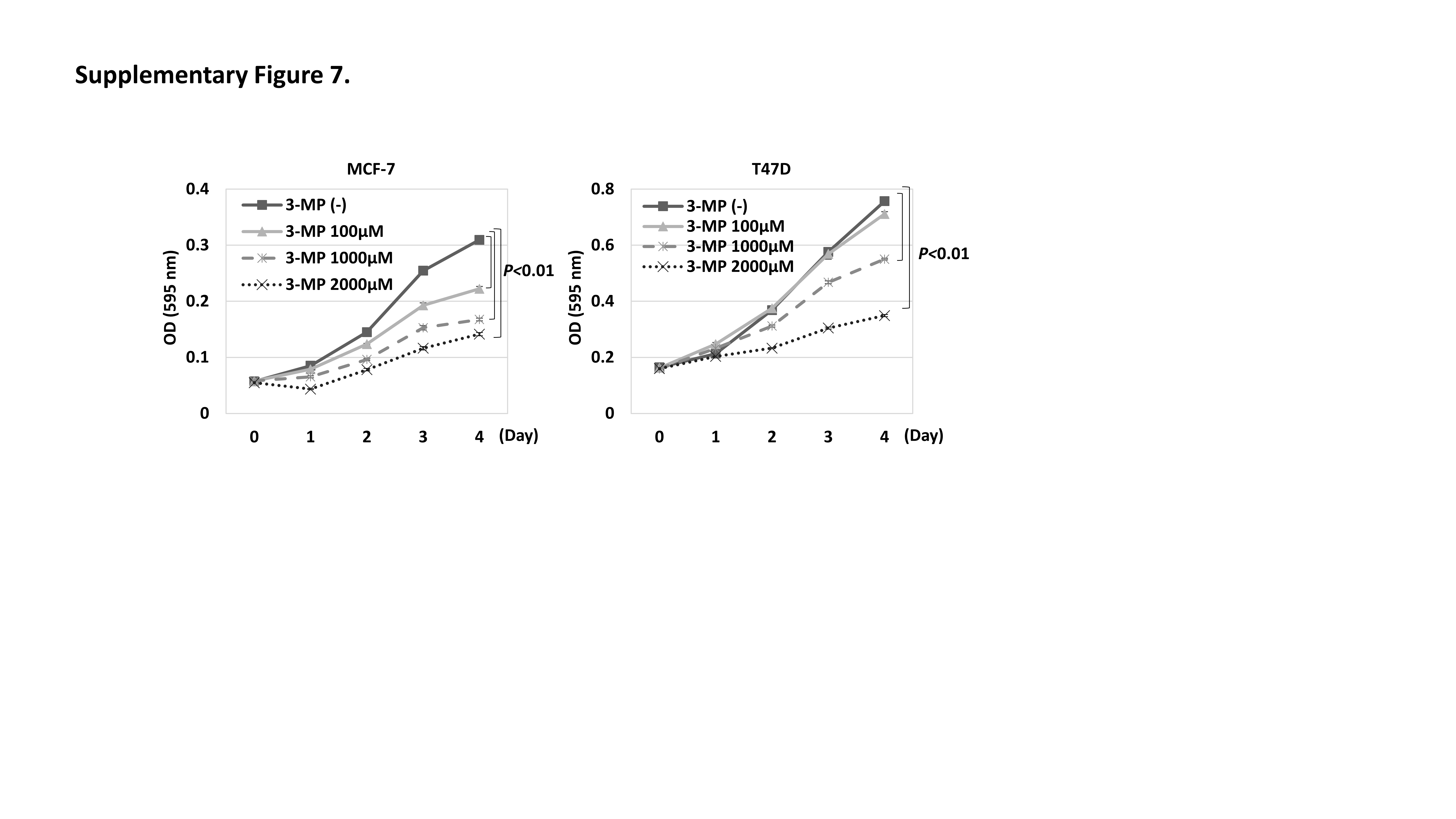

Supplement: Supplementary file 7 — Figure S7 [file CAM4-12-1588-s002.tif]

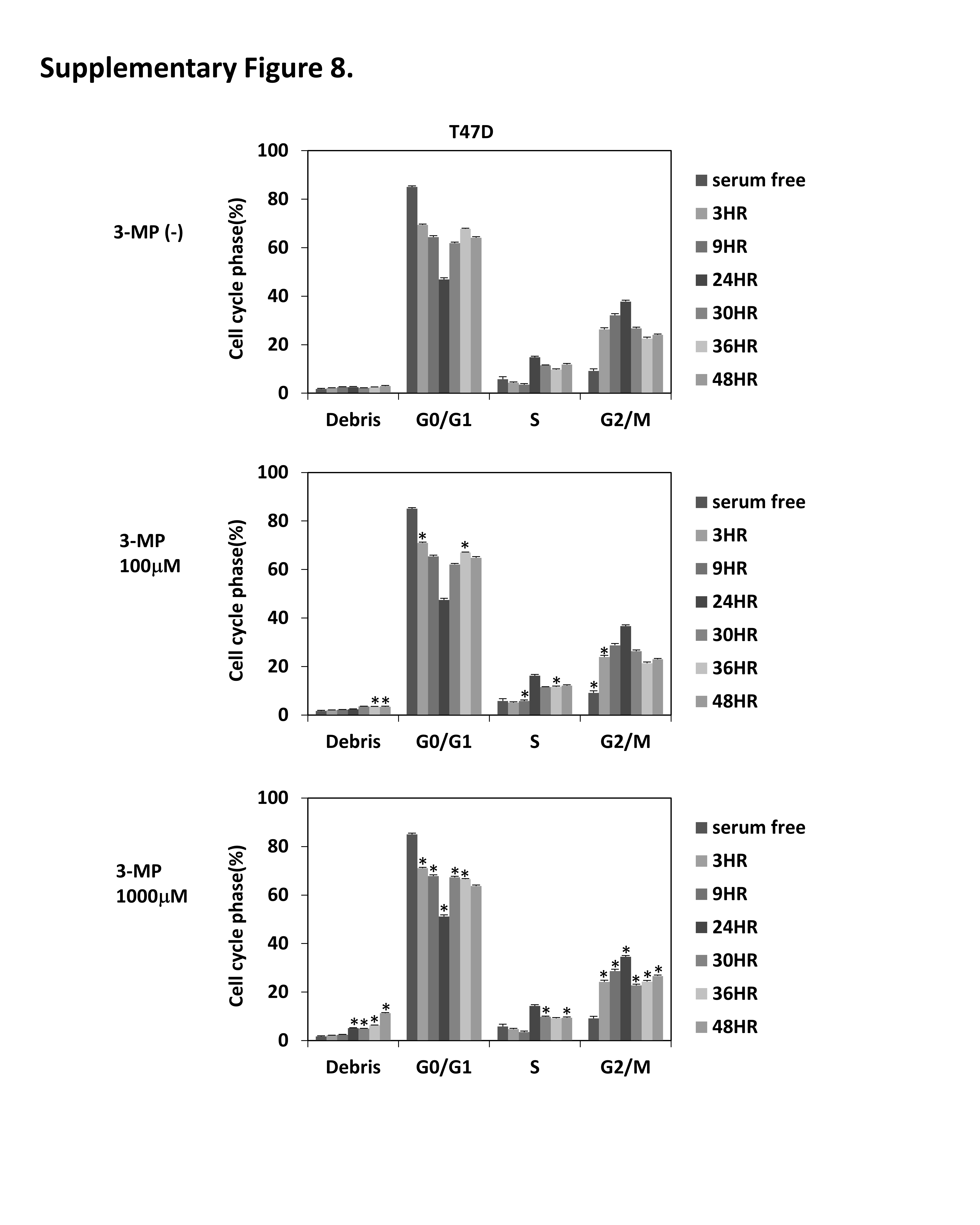

Supplement: Supplementary file 8 — Figure S8 [file CAM4-12-1588-s004.tif]

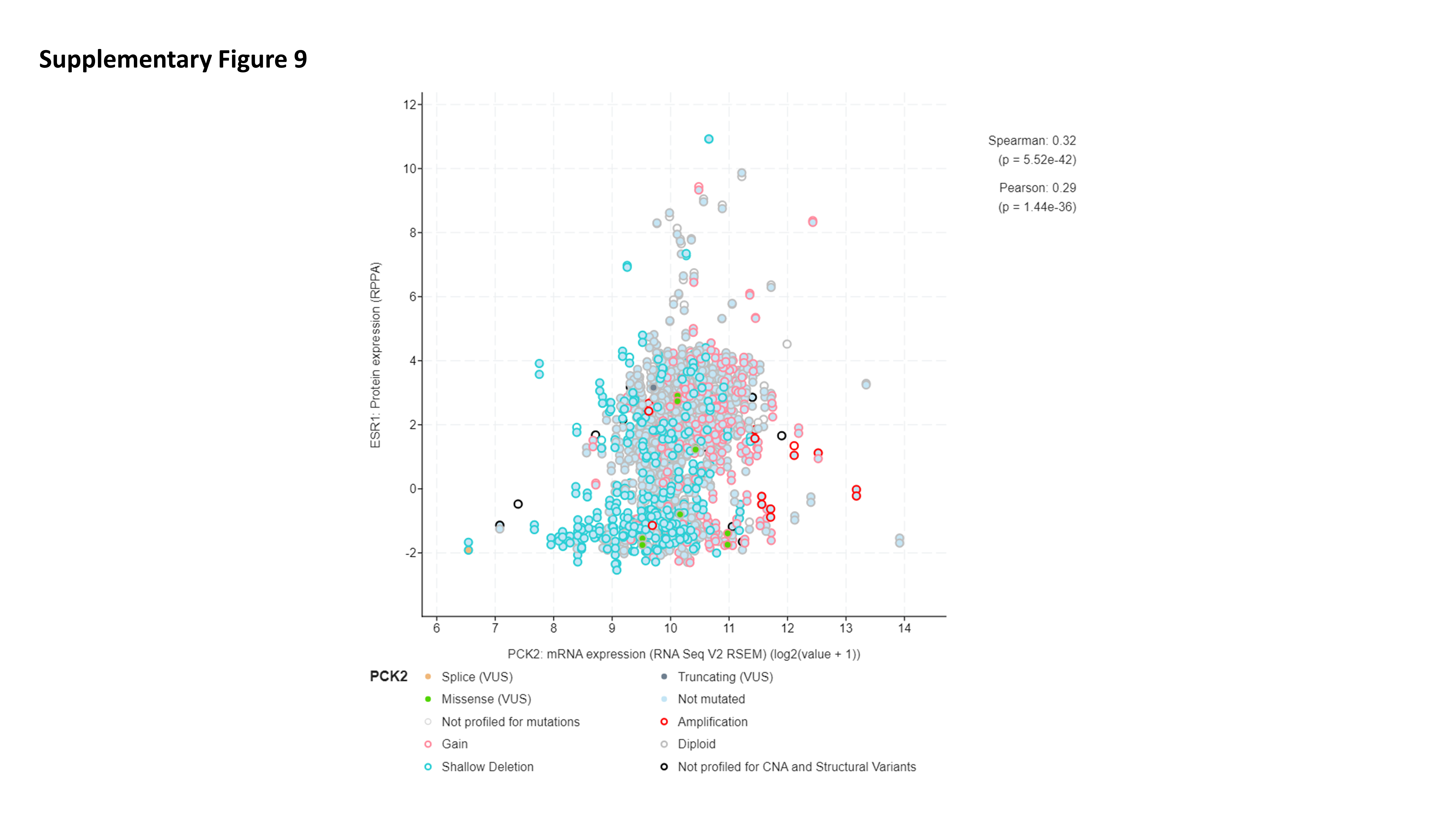

Supplement: Supplementary file 9 — Figure S9 [file CAM4-12-1588-s009.tif]
