# Supplementary material for: Mitochondrial phosphoenolpyruvate carboxykinase promotes tumor growth in estrogen receptor‐positive breast cancer via regulation of the mTOR pathway
Source: Cancer Med. 2022 Jun 27;12(2):1588–601. doi: 10.1002/cam4.4969 (PMC9883444; doi:10.1002/cam4.4969)
Supplement: Supplementary file 10 — Table S1 [file CAM4-12-1588-s008.docx]

Supplementary Table 1. The demography of the breast cancer patients

| Patient, numbers | | 177 |
| --- | --- | --- |
| Age, year, median (range) | | 52 (31 – 82) |
| Tumor size, cm, median (range) | | 2.5 (0.1 – 8.0) |
| Histological type | |  |
|  | Ductal carcinoma in situ | 4 (2%) |
|  | Invasive ductal carcinoma | 161 (90%) |
|  | Invasive lobular carcinoma | 5 (3%) |
|  | Invasive ductal + lobular carcinoma | 2 (1%) |
|  | Invasive ductal carcinoma with medullary feature | 3 (2%) |
|  | Invasive ductal carcinoma with prominent micropapillary pattern | 1 (1%) |
|  | Metaplastic carcinoma | 1 (1%) |
| Histological grade | |  |
|  | Grade I | 22 (12%) |
|  | Grade II | 66 (37%) |
|  | Grade III | 89 (51%) |
| Extensive intraductal components | |  |
|  | Negative | 117 (66%) |
|  | Positive | 60 (34%) |
| Lymphatic tumor emboli | |  |
|  | Negative | 101 (57%) |
|  | Positive | 76 (43%) |
| Fascia invasion | |  |
|  | Negative | 172 (97%) |
|  | Positive | 5 (3%) |
| Skin invasion | |  |
|  | Negative | 172 (97%) |
|  | Positive | 5 (3%) |
| Nipple invasion | |  |
|  | Negative | 138 (84%) |
|  | Positive | 26 (16%) |
| Axillary lymph node metastasis | |  |
|  | Negative | 83 (47%) |
|  | Positive | 94 (53%) |
| Positive lymph node numbers, median (range) | | 1 (0 – 43) |
| Total resected lymph node numbers, median (range) | | 18 (1 – 45) |
| Extranodal extension | |  |
|  | Negative | 127 (76%) |
|  | Positive | 41 (24%) |
| Nodal staging | |  |
|  | N0 | 83 (47%) |
|  | N1 | 56 (32%) |
|  | N2 | 15 (8%) |
|  | N3 | 23 (13%) |
| Tumor stage | |  |
|  | Tis | 5 (3%) |
|  | T1 | 44 (25%) |
|  | T2 | 115 (64%) |
|  | T3 | 10 (6%) |
|  | T4 | 3 (2%) |
| AJCC TNM stage | |  |
|  | Stage 0 | 4 (2%) |
|  | Stage I | 30 (17%) |
|  | Stage II | 102 (57%) |
|  | Stage III | 40 (23%) |
|  | Stage IV | 1 (1%) |
| Estrogen receptor | |  |
|  | Negative | 53 (30%) |
|  | Positive | 124 (70%) |
| Progesterone receptor | |  |
|  | Negative | 103 (58%) |
|  | Positive | 74 (42%) |
| HER2/neu | |  |
|  | Negative | 121 (69%) |
|  | Positive | 54 (31%) |
| Intrinsic subtype | |  |
|  | Luminal A | 43 (24%) |
|  | Luminal B1 | 54 (30%) |
|  | Luminal B2 | 30 (17%) |
|  | HER2/neu-enriched | 24 (14%) |
|  | Basal-like | 26 (15%) |
| Expression of PEPCK-M | |  |
|  | 0 | 4 (2%) |
|  | 1+ | 45 (25%) |
|  | 2+ | 93 (53%) |
|  | 3+ | 35 (20%) |
